# Metabolomic Analysis of Uremic Pruritus in Patients on Hemodialysis

**DOI:** 10.7759/cureus.86058

**Published:** 2025-06-15

**Authors:** Jehangir Afzal Mobushar, Syeda Mayedah Hussain, Waqas Amjad, Ugal Kishore, Adnan Ahmad Zafar, Muhammad Noman Qureshi, Muhammad Irfan Jamil, Iqra Naeem, Adeel Ahmed

**Affiliations:** 1 Medicine, Rashid Hospital, Dubai, ARE; 2 Internal Medicine, Services Hospital Lahore, Lahore, PAK; 3 Internal Medicine, Lahore General Hospital, Lahore, PAK; 4 Dermatology, Combined Military Hospital, Karachi, PAK; 5 Internal Medicine, Jinnah Postgraduate Medical Centre, Karachi, PAK; 6 Nephrology, Islam Medical College, Lahore, PAK; 7 Urology Department, Benaziar Bhutto Hospital, Rawalpindi Medical University, Rawalpindi, PAK; 8 Urology, Frimley Park Hospital, Camberley, GBR; 9 Nephrology, Lahore General Hospital, Lahore, PAK; 10 Psychiatry and Behavioral Sciences, Lahore General Hospital, Lahore, PAK; 11 Medicine, Lahore General Hospital, Lahore, PAK

**Keywords:** 5-d itch scale, end-stage renal disease (esrd), maintenance hemodialysis, metabolic profile, uremic pruritus

## Abstract

Background: Uremic pruritus (UP) is a prevalent and distressing complication among patients with end-stage renal disease (ESRD) receiving maintenance hemodialysis (HD). Despite its significant impact on quality of life and mortality, the underlying pathophysiology remains incompletely understood, with evidence implicating metabolic and inflammatory factors.

Objective: This study aimed to determine the prevalence of UP and assess its association with selected metabolic and inflammatory biomarkers in patients undergoing maintenance HD.

Methods: A cross-sectional observational study was conducted at the Dialysis Centre of Lahore General Hospital, a tertiary care hospital in Lahore, Pakistan. A total of 281 adult HD patients were enrolled. Pruritus was assessed using the 5-D Itch Scale, with a score ≥10 defining cases. Demographic and clinical data were recorded, and laboratory parameters including hemoglobin, calcium, phosphate, magnesium, intact parathyroid hormone (iPTH), uric acid, lipid profile, high-sensitivity C-reactive protein (hs-CRP), and white blood cell (WBC) count were analyzed. Associations were assessed using chi-square and independent samples t-tests. Multivariate logistic regression identified independent predictors of pruritus.

Results: The prevalence of UP was 82 (29.2%). Significant differences were observed between pruritic and non-pruritic groups in age, dialysis duration, and several biochemical parameters. Independent predictors of pruritus included advancing age (OR = 1.148, p < 0.001), serum phosphate (OR = 2.069, p < 0.001), uric acid (OR = 1.680, p < 0.001), iPTH (OR = 1.007, p < 0.001), hs-CRP (OR = 1.596, p = 0.001), magnesium (OR = 9.687, p = 0.020), triglycerides (OR = 1.018, p = 0.025), and HCV infection (OR = 8.098, p = 0.001). No significant associations were found with gender, diabetes, dialysis frequency, or vascular access.

Conclusion: UP in HD patients is significantly associated with metabolic and inflammatory derangements rather than demographic or dialysis-related variables. These findings underscore the importance of targeted metabolic control and inflammation modulation in managing pruritus among dialysis populations.

## Introduction

Uremic pruritus (UP) is a common and distressing symptom in patients with end-stage renal disease (ESRD), particularly those undergoing maintenance hemodialysis (HD). Its global prevalence shows considerable variation, typically ranging between 23% and over 60% [1-3]. In North America, large-scale studies conducted in the United States and Canada have reported prevalence rates ranging from 63% to 72% among incident dialysis patients [1]. In Asia, Japanese data indicate a prevalence of 61.6% among patients receiving dialysis [4]. Within Pakistan, one study from Rawalpindi reported a prevalence of 49.1% among male HD patients, while two additional studies from Peshawar and Lahore recorded prevalence rates of 23.1% in patients undergoing thrice-weekly maintenance HD [5,6].

The clinical burden of UP extends beyond physical discomfort. It is frequently generalized, persistent, and moderate to severe in intensity. The symptom has been independently associated with poor sleep quality, depressive symptoms, and reduced overall quality of life. Affected individuals report severe itching, often localized to the trunk and involving multiple body regions. Higher UP scores correlate strongly with elevated depression indices and worsened life quality scales. Importantly, UP has also been identified as an independent risk factor for increased all-cause mortality in HD patients [7-9]. While the exact pathogenesis of UP remains unclear, several metabolic and inflammatory factors have been implicated. These include disturbances in calcium, phosphate, intact parathyroid hormone (iPTH), and serum albumin levels, as well as elevations in inflammatory markers such as white blood cell (WBC) count and high-sensitivity C-reactive protein (hs-CRP) [10-13].

Given the heterogeneity of findings in the existing literature and the ongoing symptom burden in patients on HD, further exploration is needed. This study aims to investigate the association between UP and selected metabolic and inflammatory parameters, including hemoglobin, serum calcium, phosphate, magnesium, iPTH, potassium, sodium, albumin, uric acid, lipid profile, WBC count, and hs-CRP, in patients undergoing maintenance HD. By analyzing these parameters in relation to the presence or absence of pruritus, the study seeks to contribute meaningful insights into the metabolic basis of UP and support future therapeutic interventions.

## Materials and methods

This cross-sectional observational study was conducted at the Dialysis Centre of Lahore General Hospital, Lahore, over a period of six months from July 2023 to December 2024. Ethical approval for the study was obtained from the Institutional Review Board of Lahore General Hospital under reference number IRB/0133/06/2023. After obtaining informed consent, baseline demographic and clinical details were recorded through direct interviews and review of medical records. These included age, gender, duration of hemodialysis, frequency of dialysis sessions per week (twice or thrice), type of vascular access (arteriovenous fistula or central venous catheter), and history of comorbid conditions such as diabetes mellitus, hypertension, hepatitis B or C infection, and secondary hyperparathyroidism. Additional clinical information, such as history of dry skin, sleep disturbance, and prior treatment for pruritus, was also documented to aid in the clinical characterization and interpretation of symptom burden.

Eligible participants included adult patients aged 18 to 70 years of either gender who had been receiving maintenance for at least six months, either twice or thrice weekly. Patients were included in the case group if they had UP of at least four weeks' duration, confirmed using the 5-D Itch Scale with a score of ≥10 [14]. The authors have obtained permission to use the 5-D Itch Scale from the original publishers.

Patients were excluded if they had any primary dermatologic conditions known to cause itching, such as eczema, psoriasis, or scabies; documented liver disease or cholestasis; active autoimmune or malignant disorders; hypothyroidism; neurological conditions causing neuropathic itch; or significant psychiatric illness impairing symptom reporting. Additional exclusion criteria included use of antipruritic medications (such as gabapentin, antihistamines, or corticosteroids) initiated or altered within the past four weeks.

All laboratory investigations were performed using pre-dialysis venous blood samples collected under strict aseptic precautions. Samples were drawn from the arteriovenous fistula or dialysis catheter on the day of the mid-week dialysis session, at least 12 hours after the previous dialysis session and within one hour prior to the initiation of the next session, to avoid the influence of recent dialysis on serum concentrations.

The following parameters were analyzed: hemoglobin, serum calcium, phosphate, magnesium, intact parathyroid hormone (iPTH), potassium, sodium, albumin, uric acid, total white blood cell (WBC) count, high-sensitivity C-reactive protein (hs-CRP), total cholesterol, and triglycerides. Blood samples for routine biochemistry (electrolytes, uric acid, albumin, lipid profile) and complete blood count (CBC), including hemoglobin and WBC count, were collected in EDTA and plain gel tubes, respectively, and analyzed using automated hematology and chemistry analyzers (Sysmex XN-Series and Roche Cobas c501). For iPTH and hs-CRP, serum was separated and stored at 2-8°C until analysis, which was performed using electrochemiluminescence immunoassay (ECLIA) on Roche Elecsys 2010 and immunoturbidimetric assay, respectively.

Data were analyzed using IBM SPSS Statistics for Windows, Version 25.0 (released 2017, IBM Corp., Armonk, NY). The Chi-square test was used to assess associations between categorical variables, while the independent samples t-test was applied to compare continuous variables between pruritic and non-pruritic groups. Multivariate logistic regression was performed to identify independent predictors of uremic pruritus. A p-value of <0.05 was considered statistically significant.

## Results

Out of 281 patients undergoing maintenance hemodialysis, UP was observed in 82 individuals, indicating a prevalence of 29.2%. Age group analysis revealed higher pruritus prevalence among patients aged 41-70 years, but the association was not statistically significant (χ² = 2.711, p = 0.100; OR = 1.706, 95% CI: 0.899-3.236). No significant gender-based difference was found (χ² = 1.316, p = 0.251; OR = 1.352, 95% CI: 0.807-2.264). Socioeconomic status showed a statistically significant relationship with pruritus (χ² = 17.364, p < 0.001). Dialysis frequency, diabetes mellitus, hypertension, and vascular access type were not significantly associated with pruritus, with p-values of 0.686, 0.120, 0.070, and 0.766, respectively. However, hepatitis C virus infection demonstrated a significant association with pruritus (χ² = 24.608, p < 0.001), with an OR of 4.290 (95% CI: 2.356-7.814), identifying it as a strong independent predictor (Table 1).

**Table 1:** Association between sociodemographic and clinical variables and uremic pruritus in hemodialysis patients (n = 281). Table showing the association between sociodemographic and clinical variables and uremic pruritus in hemodialysis patients (n = 281), analyzed using the Chi-square test and univariate logistic regression. Odds ratios (OR) with 95% confidence intervals (CI) are presented. Reference categories used for OR calculations are: age group 18–40 years, female, high socioeconomic status, twice-weekly dialysis, non-diabetic status, non-hypertensive status, AV fistula access, HBV-negative, and HCV-negative. Abbreviations: OR = odds ratio, CI = confidence interval, CVC = central venous catheter, AV = arteriovenous, HBV = hepatitis B virus, HCV = hepatitis C virus

Variable	Subcategory	Uremic pruritus: Yes (n = 82)	Uremic pruritus: No (n = 199)	Chi-square (χ²)	OR (95% CI)	p-value
Age group	41–70 years	67 (81.7%)	144 (72.4%)	2.711	1.706 (0.899–3.236)	0.100
	18–40 years	15 (18.3%)	55 (27.6%)			
Gender	Male	40 (48.8%)	112 (56.3%)	1.316	1.352 (0.807–2.264)	0.251
	Female	42 (51.2%)	87 (43.7%)			
Socioeconomic status	Low	50 (61.0%)	69 (34.7%)	17.364	-	<0.001
	Middle	20 (24.4%)	94 (47.2%)			
	High	12 (14.6%)	36 (18.1%)			
Dialysis frequency	Twice weekly	70 (85.4%)	166 (83.4%)	0.164	0.862 (0.421–1.767)	0.686
	Thrice weekly	12 (14.6%)	33 (16.6%)			
Diabetes mellitus	Yes	29 (35.4%)	52 (26.1%)	2.414	1.547 (0.890–2.687)	0.120
	No	53 (64.6%)	147 (73.9%)			
Hypertension	Yes	63 (76.8%)	131 (65.8%)	3.287	0.581 (0.322–1.049)	0.070
	No	19 (23.2%)	68 (34.2%)			
Vascular access	CVC	22 (26.8%)	50 (25.1%)	0.088	1.093 (0.609–1.960)	0.766
	AV fistula	60 (73.2%)	149 (74.9%)			
HBV status	Positive	8 (9.8%)	18 (9.0%)	0.035	1.087 (0.453–2.609)	0.852
	Negative	74 (90.2%)	181 (91.0%)			
HCV status	Positive	33 (40.2%)	27 (13.6%)	24.608	4.290 (2.356–7.814)	<0.001
	Negative	49 (59.8%)	172 (86.4%)			

Patients with UP showed significantly higher age, dialysis duration, hemoglobin, phosphorus, uric acid, intact PTH, hs-CRP, magnesium, and triglyceride levels compared to those without pruritus. No significant differences were observed for calcium, albumin, sodium, creatinine, cholesterol, or potassium. These results suggest a strong metabolic and inflammatory contribution to the pathogenesis of UP (Table 2).

**Table 2:** Comparison of continuous biochemical and clinical parameters between hemodialysis patients with and without uremic pruritus (n = 281). This table compares the means of clinical and biochemical variables between patients with and without uremic pruritus using independent samples t-tests. Abbreviations: PTH = parathyroid hormone; hs-CRP = high-sensitivity C-reactive protein; WBC = white blood cell count; SD = standard deviation; CI = confidence interval

Parameter	Uremic pruritus: Yes (mean ± SD) (n = 82)	Uremic pruritus: No (mean ± SD) (n = 199)	Mean difference	t-value	95% CI (lower–upper)	p-value
Age (years)	51.22 ± 9.94	42.76 ± 8.58	-8.461	-7.166	-10.785 to -6.136	<0.001
Dialysis duration (months)	27.44 ± 15.86	23.31 ± 10.48	-4.132	-2.563	-7.306 to -0.959	0.011
Hemoglobin (g/dL)	10.87 ± 1.39	9.86 ± 2.15	-1.0096	-3.923	-1.516 to -0.503	<0.001
Serum calcium (mg/dL)	8.11 ± 0.76	8.13 ± 0.88	0.0201	0.182	-1.977 to 2.379	0.856
Serum phosphorus (mg/dL)	6.39 ± 1.81	4.97 ± 1.04	-1.4132	-8.228	-1.751 to -1.075	<0.001
Serum uric acid (mg/dL)	6.35 ± 2.14	5.65 ± 1.82	-0.6980	-2.773	-1.193 to -0.202	0.006
Intact PTH (pg/mL)	316.89 ± 253.22	201.89 ± 106.03	-115.00	-5.374	-157.13 to -72.87	<0.001
Serum albumin (g/dL)	3.91 ± 0.38	3.97 ± 0.36	0.0554	1.147	-0.0397 to 0.1506	0.252
WBC count (×10³/μL)	7.33 ± 1.56	7.90 ± 1.69	0.5648	2.605	0.1380 to 0.9915	0.010
hs-CRP (mg/L)	4.66 ± 2.21	3.10 ± 1.61	-1.5557	-6.577	-2.021 to -1.090	<0.001
Serum magnesium (mg/dL)	2.10 ± 0.24	1.96 ± 0.32	-0.1472	-3.764	-0.2241 to -0.0702	<0.001
Serum potassium (mEq/L)	4.58 ± 0.84	4.40 ± 0.69	-0.1821	-1.886	-0.3721 to 0.0079	0.060
Serum sodium (mEq/L)	144.00 ± 7.22	144.29 ± 7.77	0.2864	0.287	-1.6809 to 2.2538	0.775
Serum creatinine (mg/dL)	5.30 ± 1.51	5.35 ± 1.54	0.0483	0.240	-0.3478 to 0.4445	0.810
Triglycerides (mg/dL)	177.76 ± 33.40	155.97 ± 33.63	-21.78	-4.945	-30.45 to -13.11	<0.001
Total cholesterol (mg/dL)	203.71 ± 59.01	195.49 ± 43.62	-8.22	-1.289	-20.77 to 4.333	0.198

The final multivariate analysis identified advancing age (OR = 1.148), serum phosphate (OR = 2.069), uric acid (OR = 1.680), intact PTH (OR = 1.007), hs-CRP (OR = 1.596), magnesium (OR = 9.687), and triglycerides (OR = 1.018) as significant independent predictors of UP. HCV positivity (OR = 8.098) and low socioeconomic status (OR = 0.150) were also strongly associated. The model demonstrated good fit (Nagelkerke R² = 0.756) and classification accuracy of 90% (χ² = 212.222, p < 0.001) (Table 3).

**Table 3:** Multivariate logistic regression analysis of predictors for uremic pruritus among hemodialysis patients (n = 281). The regression coefficient (B) represents the direction and magnitude of association between each predictor and pruritus, while the Wald statistic tests the null hypothesis that the coefficient equals zero (i.e., no association). A higher Wald value indicates a stronger contribution to the model. Reference categories used in the model are as follows: younger age group (per year increase), lower phosphate, uric acid, and CRP levels, low serum magnesium and triglycerides, no HCV infection, and high socioeconomic status (SES). Abbreviations: OR = odds ratio, CI = confidence interval, hs-CRP = high-sensitivity C-reactive protein, PTH = parathyroid hormone, DM = diabetes mellitus, HTN = hypertension, SES = socioeconomic status, HCV = hepatitis C virus, HBV = hepatitis B virus, AV = arteriovenous, CVC = central venous catheter

Variable	B	Wald	p-value	Adjusted OR (Exp(B))	95% CI for OR
Age (years)	0.138	18.846	<0.001	1.148	1.079-1.222
Dialysis duration (months)	0.025	1.188	0.276	1.025	0.980-1.073
Hemoglobin (g/dL)	0.320	3.783	0.052	1.377	0.998-1.900
Serum phosphate (mg/dL)	0.727	13.708	<0.001	2.069	1.408-3.040
Serum uric acid (mg/dL)	0.519	12.583	<0.001	1.680	1.261-2.239
Intact PTH (pg/mL)	0.007	13.441	<0.001	1.007	1.003-1.010
WBC count (×10³/μL)	-0.049	0.071	0.790	0.952	0.664-1.365
hs-CRP (mg/L)	0.468	12.200	0.001	1.596	1.228-2.075
Serum magnesium (mg/dL)	2.271	5.373	0.020	9.687	1.420-66.076
Serum potassium (mEq/L)	0.743	3.538	0.060	2.102	0.969-4.559
Triglycerides (mg/dL)	0.018	5.006	0.025	1.018	1.002-1.034
Total cholesterol (mg/dL)	-0.003	0.215	0.643	0.997	0.986-1.009
DM (Yes vs. No)	-0.673	1.395	0.238	0.510	0.167-1.559
HTN (Yes vs. No)	-1.081	3.540	0.060	0.339	0.110-1.046
SES: Low vs. High	-1.896	8.564	0.003	0.150	0.042-0.535
SES: Middle vs. High	-1.238	3.160	0.075	0.290	0.074-1.135
HCV	2.092	11.829	0.001	8.098	2.459-26.673
HBV	0.330	0.167	0.683	1.390	0.286-6.748

## Discussion

In this study of 281 hemodialysis patients, the prevalence of UP was 29.2%, aligning with regional estimates but lower than global figures ranging from 40% to 60% [15,16]. No significant association was observed with gender or diabetes status, indicating that UP spans across demographic groups. However, patients with pruritus were significantly older (mean age 51.22 vs. 42.76 years; p < 0.001), and age remained an independent predictor in multivariate analysis (OR = 1.148; 95% CI: 1.079-1.222; p < 0.001), suggesting cumulative exposure to the uremic milieu as a contributing factor. While prior studies found no age-related differences [12,17], our findings align with data indicating increased pruritus in older individuals [18].

Socioeconomic status (SES) also showed a significant relationship. Although low SES patients were overrepresented among those with pruritus (61.0%), multivariate analysis revealed they had lower odds compared to high SES groups (OR = 0.150; 95% CI: 0.042-0.535; p = 0.003). This inverse association may reflect complex behavioral or healthcare access patterns. Global studies have demonstrated wide variability in pruritus prevalence, influenced by resource disparities, dialysis quality, and health system differences [15,16].

Hepatitis C virus (HCV) infection emerged as a strong independent predictor (OR = 8.098; 95% CI: 2.459-26.673; p = 0.001), with 40.2% of pruritic patients HCV-positive. This aligns with previous findings where HCV was linked to increased pruritus burden [19-21]. Mechanistically, HCV-related cholestasis, cytokine dysregulation, and cutaneous nerve sensitization may explain this association [19]. Conversely, HBV showed no significant association (OR = 1.087; p = 0.852), consistent with DOPPS and other literature [16,21]. These findings highlight the clinical utility of HCV screening in pruritus assessment among dialysis patients.

In this cohort, hypertension was highly prevalent among both pruritic and non-pruritic patients (76.8% vs. 65.8%), yet the association did not reach statistical significance (p = 0.070) and was not retained in multivariate analysis (OR = 0.339; p = 0.060). These findings align with previous studies reporting no independent link between hypertension and UP [22,23]. Although microvascular inflammation and oxidative stress have been proposed as possible mediators, such mechanisms remain speculative and lack consistent clinical validation [24].

Similarly, diabetes mellitus did not demonstrate a significant association with pruritus in either univariate or adjusted analyses (p = 0.120; OR = 0.510). This supports findings from Zhao et al. (2021) and the DOPPS study, which concluded that diabetes may coexist with pruritus but is unlikely to be causative [12,16]. While peripheral neuropathy might dampen itch perception and chronic inflammation might amplify it, neither theory has been conclusively supported [22,23].

Dialysis frequency showed no significant relationship with pruritus (p = 0.686), indicating that the number of weekly sessions alone may not be sufficient to alleviate symptom burden. By contrast, dialysis adequacy, as highlighted in prior studies, appears to play a more pivotal role in pruritus mitigation. The longer dialysis duration observed among pruritic patients (p = 0.011) further supports the role of cumulative toxin exposure in the development of symptoms [16,17,25].

Hyperphosphatemia demonstrated a strong and statistically significant association with UP in this cohort. Patients experiencing pruritus had markedly elevated serum phosphate levels compared to those without pruritus, and multivariate logistic regression confirmed phosphate as an independent predictor (OR = 2.069; 95% CI: 1.408-3.040; p < 0.001). These findings support previous data from the DOPPS study [16], where phosphorus was identified as a predictor of moderate-to-severe pruritus, and are consistent with Narita et al. linked elevated calcium-phosphate product to higher pruritus prevalence [10]. Histological evidence by Momose et al. (2017) further substantiates the deposition of calcium-phosphate in pruritic skin [26]. By contrast, some other studies found no such association, possibly due to stricter phosphate control in their cohorts [12,27]. Intact parathyroid hormone (iPTH) was also significantly associated with pruritus, with a 115 pg/mL mean difference between groups and independent predictive value in multivariate analysis (OR = 1.007; 95% CI: 1.003-1.010; p < 0.001). This echoes prior research suggesting a role for secondary hyperparathyroidism in pruritus pathogenesis [10,26]. However, its influence may be attenuated when inflammation is accounted for [16,26].

Inflammatory status, as measured by hs-CRP, showed the strongest association. Pruritic patients had significantly higher hs-CRP levels, and multivariate analysis confirmed CRP as a robust predictor (OR = 1.596; 95% CI: 1.228-2.075; p = 0.001). Previous studies support this finding, highlighting CRP as both a biomarker and mediator of itch through pro-inflammatory cytokines [12,13,21]. Although hemoglobin was higher in pruritic patients in univariate analysis, the association was not significant after adjustment (OR = 1.377; p = 0.052). Prior findings are inconsistent-Sukul et al. (2020) reported lower hemoglobin [23], while others suggested no direct relationship [16,22]. It remains possible that hemoglobin reflects broader inflammatory or nutritional status rather than acting as an independent factor.

In the present study, serum magnesium emerged as a significant independent predictor of UP (OR = 9.687; 95% CI: 1.420-66.076; p = 0.020), with higher levels noted among pruritic patients. This finding stands in contrast to an earlier study, which reported no independent association after adjusting for confounders [24]. However, another study hypothesized that magnesium, along with other divalent ions, may precipitate in the dermis and provoke cutaneous irritation and may offer partial mechanistic plausibility [28]. Further mechanistic studies are required to validate this association. Serum potassium levels, while slightly elevated in the pruritic group, showed no statistically significant association (p = 0.060), and the observed borderline odds ratio (OR = 2.102) remained inconclusive. Previous studies similarly did not support potassium as a relevant factor in CKD-aP, reinforcing its limited pathophysiological relevance [12,17,21].

Serum triglycerides were independently associated with pruritus (OR = 1.018; 95% CI: 1.002-1.034; p = 0.025), indicating a potential inflammatory-metabolic link. Although Kimata et al. (2016) found elevated lipid-related metabolites in pruritic patients and Ko et al. (2015) linked triglycerides to systemic inflammation [25,29]. The modest effect size in this study suggests that triglycerides may serve more as a surrogate marker than a direct pruritic factor.

This study highlights key clinical implications for managing UP, emphasizing the role of routine screening and targeted interventions focused on modifiable metabolic and inflammatory factors. Optimizing phosphate, iPTH, and triglyceride levels, along with managing systemic inflammation and HCV infection, may offer symptomatic relief. However, the study’s cross-sectional design limits causal inference, and unmeasured confounders, such as vitamin D levels or pruritogenic medications, may influence findings. The single-center setting may also limit generalizability to broader populations. Despite these limitations, the findings offer valuable insights that can inform personalized, multifaceted approaches to alleviate pruritus in the hemodialysis population.

## Conclusions

This study concluded that UP is influenced predominantly by metabolic and inflammatory parameters rather than demographic or dialysis-related factors. Advancing age, elevated serum phosphate, uric acid, iPTH, hs-CRP, magnesium, and triglyceride levels were identified as independent predictors of pruritus. HCV infection also emerged as a strong contributor. By contrast, gender, diabetes, hypertension, dialysis frequency, vascular access, and HBV status showed no significant associations. These findings highlight the multifactorial nature of pruritus and reinforce the importance of addressing systemic inflammation and mineral metabolism in clinical management.
